# The genome of the intracellular bacterium of the coastal bivalve, *Solemya velum*: a blueprint for thriving in and out of symbiosis

**DOI:** 10.1186/1471-2164-15-924

**Published:** 2014-10-23

**Authors:** Oleg Dmytrenko, Shelbi L Russell, Wesley T Loo, Kristina M Fontanez, Li Liao, Guus Roeselers, Raghav Sharma, Frank J Stewart, Irene LG Newton, Tanja Woyke, Dongying Wu, Jenna Morgan Lang, Jonathan A Eisen, Colleen M Cavanaugh

**Affiliations:** Department of Organismic and Evolutionary Biology, Harvard University, 16 Divinity Avenue, 4081 Biological Laboratories, Cambridge, MA 02138 USA; Department of Civil and Environmental Engineering, Massachusetts Institute of Technology, 15 Vassar Street, Cambridge, MA 02139 USA; SOA Key Laboratory for Polar Science, Polar Research Institute of China, Shanghai, 200136 China; Microbiology & Systems Biology Group, TNO, Utrechtseweg 48, Zeist, Utrecht, 3704 HE The Netherlands; School of Biology, Georgia Institute of Technology, Atlanta, GA 30332-0230 USA; Department of Biology, Indiana University, 1001 East 3rd Street, Jordan Hall, Bloomington, IN 47405 USA; DOE Joint Genome Institute, 2800 Mitchell Drive, Walnut Creek, CA 94598 USA; UC Davis Genome Center, 451 East Health Sciences Drive, Davis, CA 95616-8816 USA

**Keywords:** Symbiosis, Chemosynthesis, Sulfur oxidation, Respiratory flexibility, H^+^/Na^+^ -membrane cycles, Calvin cycle, Pyrophosphate-dependent phosphofructokinase, Heterotrophy, Motility, Mobile genetic elements

## Abstract

**Background:**

Symbioses between chemoautotrophic bacteria and marine invertebrates are rare examples of living systems that are virtually independent of photosynthetic primary production. These associations have evolved multiple times in marine habitats, such as deep-sea hydrothermal vents and reducing sediments, characterized by steep gradients of oxygen and reduced chemicals. Due to difficulties associated with maintaining these symbioses in the laboratory and culturing the symbiotic bacteria, studies of chemosynthetic symbioses rely heavily on culture independent methods. The symbiosis between the coastal bivalve, *Solemya velum*, and its intracellular symbiont is a model for chemosynthetic symbioses given its accessibility in intertidal environments and the ability to maintain it under laboratory conditions*.* To better understand this symbiosis, the genome of the *S. velum* endosymbiont was sequenced.

**Results:**

Relative to the genomes of obligate symbiotic bacteria, which commonly undergo erosion and reduction, the *S. velum* symbiont genome was large (2.7 Mb), GC-rich (51%), and contained a large number (78) of mobile genetic elements. Comparative genomics identified sets of genes specific to the chemosynthetic lifestyle and necessary to sustain the symbiosis. In addition, a number of inferred metabolic pathways and cellular processes, including heterotrophy, branched electron transport, and motility, suggested that besides the ability to function as an endosymbiont, the bacterium may have the capacity to live outside the host.

**Conclusions:**

The physiological dexterity indicated by the genome substantially improves our understanding of the genetic and metabolic capabilities of the *S. velum* symbiont and the breadth of niches the partners may inhabit during their lifecycle.

**Electronic supplementary material:**

The online version of this article (doi:10.1186/1471-2164-15-924) contains supplementary material, which is available to authorized users.

## Background

Symbiosis is one of the major driving forces of evolutionary adaptation. Chloroplasts and mitochondria are examples of ancient symbiotic partnerships which played key roles in the emergence and diversification of eukaryotic life on Earth [1]. Bacteria have been found in symbioses with organisms as diverse as plants, insects, marine invertebrates, and protists [2–5], expanding metabolic capabilities of the partners and allowing them to occupy otherwise unavailable ecological niches. Despite the ubiquity of such mutualistic associations and their importance to health and the environment, studies of many host-associated microorganisms have been complicated by difficulties in both the maintenance of symbiotic organisms in culture and the inability to genetically manipulate them. However, progress in culture-independent techniques has allowed for rapid advances in understanding symbiosis diversity, evolution, genetics, and physiology [6–8].

Symbioses between chemoautotrophic bacteria and invertebrates are ubiquitous in reducing marine habitats, such as deep-sea hydrothermal vents and coastal sediments. In these environments, the symbiotic bacteria derive energy by oxidizing reduced inorganic molecules (e.g., sulfide) and fix carbon dioxide for biomass production. Their hosts have evolved behavioral, physiological, and biochemical adaptations for capturing and delivering the required electron donors and acceptors to the symbionts. In return, these invertebrates obtain their nutrition from bacterial chemosynthesis [5, 9].

*Solemya velum* and its endosymbionts is one of the best-described chemoautotrophic symbioses. The host, a protobranch bivalve, lives in coastal nutrient-rich sediments where it builds Y-shaped burrows that span the oxic-anoxic interface, allowing access to both reduced inorganic sulfur as an energy source and oxygen for use as a terminal oxidant [10]. The symbionts, which constitute a single 16S rRNA phylotype of γ-proteobacteria [11], are localized to specialized epithelial cells (bacteriocytes) in the gills, separated from the cytoplasm by a peribacterial membrane. Using energy from the oxidation of sulfide, the symbionts fix CO_2_ via the Calvin-Benson-Bassham Cycle [12, 13]. Primary production in the symbionts sustains the host, which has only a rudimentary gut and cannot effectively filter-feed [14, 15]. Many key properties of this symbiosis still remain to be characterized, including the exchange of metabolites and signals between the symbiont and the host and the mechanism of symbiont acquisition at each new host generation (i.e., symbiont transmission mode).

The mode by which *S. velum* acquires its symbionts has important implications for understanding symbiont genome evolution. Symbiont-specific genes have been amplified from the host ovarian tissue of both *S. velum* and its congener, *S. reidi*[16, 17], raising the hypothesis that symbionts are transmitted maternally (vertically) between successive host generations via the egg. Vertical transmission has also been inferred in deep-sea clams of the Vesicomyidae [18, 19], in which symbionts have a reduced genome size (1.2 Mb) and appear to be obligately associated with their host [20–23]. In vesicomyid symbioses, host and symbiont phylogenies are largely congruent, a pattern consistent with vertical symbiont transmission [24]. Nonetheless, instances of lateral symbiont movement among some vesicomyids have been inferred based on decoupling of symbiont and host evolutionary trajectories [25], bringing diverse symbiont strains into contact and creating opportunities for symbiont genome evolution via recombination [26, 27]. In the Solemyidae, on the other hand, symbionts of different *Solemya* species are scattered across phylogenetic clades (i.e., polyphyly), indicating distinct evolutionary origins relative to the monophyly of the hosts [5, 28]. A preliminary analysis was unable to definitively resolve the extent of genetic coupling between the *S. velum* host and its symbionts in populations along the southern New England coast [26]. These patterns may be the result of a physical decoupling of symbiont and host lineages, possibly due to lateral symbiont transmission between hosts.

It is therefore possible that transmission in solemyid symbioses, as in vesicomyids, involves a combination of both vertical passage through the maternal germ line and lateral acquisition of symbionts from the environment or other co-occurring host individuals. Such a mixed transmission mode could strongly impact symbiont genome evolution by creating opportunities for lateral gene transfer, relieving the constraints of genetic bottlenecks imposed by strict vertical transmission [29, 30], and imposing selective pressures for the maintenance of diverse functions in the symbiont genome that would mediate survival outside the host. The genome of the *S. velum* symbiont will provide insights into the transmission mode of this symbiont, define a framework for examining its physiological adaptations, and supply a reference sequence for future studies of the ecology and evolution of solemyid symbionts.

Here we present an analysis of the genome from the *S. velum* symbiont. First, genes that encode core metabolic functions are discussed. Emphasis is placed on bioenergetics, autotrophy, heterotrophy, and nitrogen metabolism, which indicate metabolic potential beyond strict chemolithoautotrophy. Genes encoding cellular functions that pertain to the symbiotic lifestyle are also analyzed. A special focus is on the processes, such as membrane transport, sensing, and motility that may be involved in interactions of the symbiont with the host and the environment. Wherever appropriate, the gene content is compared to that of free-living and host-associated bacteria, in particular the intracellular chemosynthetic symbionts of the vesicomyid clams, *Calyptogena magnifica*[22] and *Calyptogena okutanii*[20], the vestimentiferan tubeworms, *Riftia pachyptila*[31] and *Tevnia jerichona*[32], the scaly-foot snail, *Crysomollon squamiferum,*[33] and the marine oligochaete worm, *Olavius algarvensis,*[34]. This comprehensive analysis defines the *S. velum* symbiont as a metabolically versatile bacterium adapted to living inside the host but also potentially capable of survival on the outside. It informs attempts to culture the symbionts and generates multiple intriguing hypotheses that now await experimental validation.

## Results and discussion

### General genome features

The genome of the *S. velum* symbiont consists of 10 non-overlapping scaffolds, totaling 2,702,453 bp, with an average G + C content of 51%. The three largest scaffolds (1.21 Mb, 0.89 Mb, 0.54 Mb) contain 97.8% of the total genomic sequence and 98.4% of the predicted genes (Additional file 1: Table S1). Assembly of the scaffolds into a closed genome was prevented by stretches of single nucleotides or groups of a few nucleotides repeated up to 70 times that could not be spanned. However, the high depth of sequence coverage and the presence of all 31 core bacterial phylogenetic gene markers [35] suggest that most gene-coding regions were detected in the analysis. Nevertheless, as the genome is not closed, a definitive list of all symbiont genes could not be made.

An overview of the *S. velum* symbiont genome compared to selected symbiotic and free-living γ-proteobacteria, including other thiotrophs, is presented in Table 1. Briefly, 90.7% of the genome sequence encodes 2,757 genes, on average 885 bp long. 2,716 (98.5%) genes are protein-coding. Function was predicted for 1,988 (72.1%) of all the genes, while 769 (27.9%) were identified as encoding hypothetical proteins. 382 genes (13.8%) have one or more paralog in the genome, with the largest paralogous group encoding transposases associated with mobile elements. The genome contains a single ribosomal RNA (rRNA) operon and 38 transfer RNAs (tRNA) corresponding to the 20 standard proteinogenic amino acids. Due to the wobble base-pairing [36], tRNAs for each given amino acid can pair with any codon in the genome for that amino acid (Additional file 2: Table S2).

**Table 1:** **General genome features of the** ***S. velum*** **symbiont in comparison to other γ-proteobacteria**

	***Solemya velum*** endosymbiont	***Riftia pachyptila*** endosymbiont *	***Calyptogena magnifica*** endosymbiont	***Calyptogena okutanii*** endosymbiont	***Buchnera aphidicola*** APS	Ca. ***Carsonella ruddii*** PV	***Thiomicrospira crunogena*** XCL-2	***Allochromatium vinosum*** DSM 180	***Escherichia coli*** K12 DH1, ATCC 33849
**Size, mb**	**2.70**	3.20	1.20	1.02	0.65	0.16	2.40	3.60	4.63
**G + C%**	**51.0**	57.9	34.0	31.6	26.4	16.6	43.1	64.3	50.8
**ORFs**	**2757**	4182	1118	981	615	213	2263	3317	4273
**Average ORF length, bp**	**885**	354	874	897	935	737	974	1005	940
**Percent coding**	**90.7**	69.8	79.8	85.9	87.6	97.3	90.5	90.6	86.6
**rRNA operons (16S-23S-5S)**	**1**	1	1	1	1	1	3	3	7
**tRNA genes**	**38**	32	36	36	32	28	43	51	88
**Proteins with predicted function**	**1988**	2218	932	838	561	113	1785	2505	3506
**Hypothetical and uncharacterized conserved proteins**	**769**	3693	175	253	106	46	689	924	833
**ORFs in paralogous families**	**382**	292	27	19	7	0	159	413	794
**Pseudogenes**	**0**	0	100	2	1	0	8	81	178
**Sigma factors**	**9**	4	2	2	2	0	6	6	7
**Mobile elements**	**78**	10	0	0	0	0	10	19	39
	Symbiont	Symbiont	Symbiont	Symbiont	Symbiont	Symbiont	Free-living	Free-living	Free-living

The comparison includes genomes of the chemosynthetic symbionts of *R. pahyptila*, *C. magnifica*, and *C. okutanii*; a symbiont of psyllids (the smallest sequenced genome), *Carsonella ruddii*; an α-proteobacterial aphid symbiont, *B. aphidicola*; free-living sulfur-oxidizers, *T. crunogena* and *A. vinosum*, and enterobacterium *E. coli*. *NCBI Accession PRJNA16744 and PRJNA72967.

**Figure 1:**
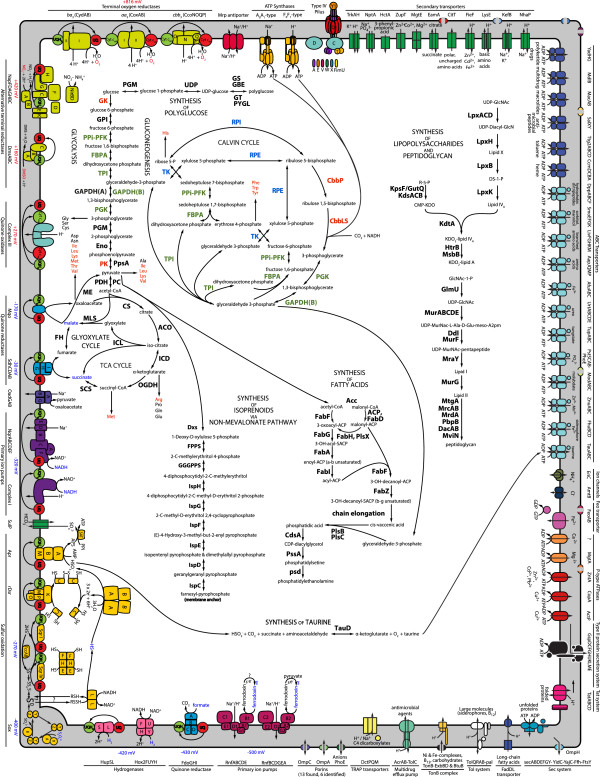
**Predicted model of the** ***S. velum*** **symbiont cell.** The diagram, based on the gene annotation of the symbiont genome, depicts key functional systems and metabolic pathways: sulfur oxidation, electron transport, ATP synthases, CO_2_-fixation via the Calvin Cycle, gluconeogenesis, polyglucose synthesis, glycolysis, TCA and glyoxylate cycles, synthesis of amino acids, fatty acids, lipids, isoprenoids via non-mevalonate pathway, and the cell wall, solute transporters, protein secretion systems, and the type IV pilus. Different protein categories are color-coded and the individual subunits indicated by shape symbols. The direction of substrate transport across the membrane is shown with arrows. Components of the electron transport chain are arranged from the lowest to the highest electronegativity of the electron donors (blue) and acceptors (red). The corresponding electronegativity values are listed next to the respective enzymes. Enzymes shared between glycolysis, gluconeogenesis, and the Calvin cycle are designated in green. Enzymes unique to these pathways are designated in red. Enzymes shared between the Calvin cycle and the pentose phosphate cycle are designated in blue. Amino acids which may be essential for the host are designated in red. Speculated pathways are designated with a question mark. The abbreviations used, the respective full gene product names, and the corresponding NCBI protein ID references are listed in Additional file 3: Table S3.

**Figure 2:**
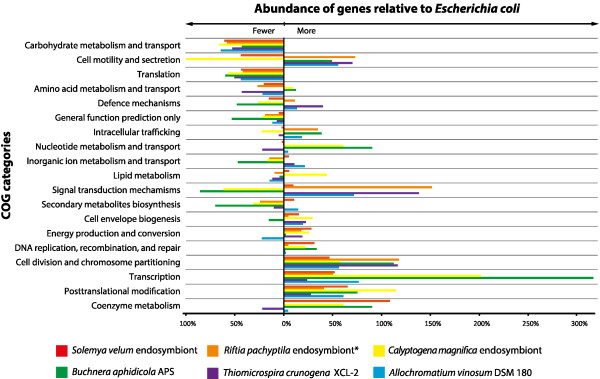
**Comparison of the COG categories between the** ***S. velum*** **symbiont and selected symbiotic and free-living bacteria.** The percentage of genes in each category is normalized to the percentage of those COG categories in the genome of *E. coli* K12 DH1, ATCC 33849. *NCBI accession PRJNA16744.

**Figure 3:**
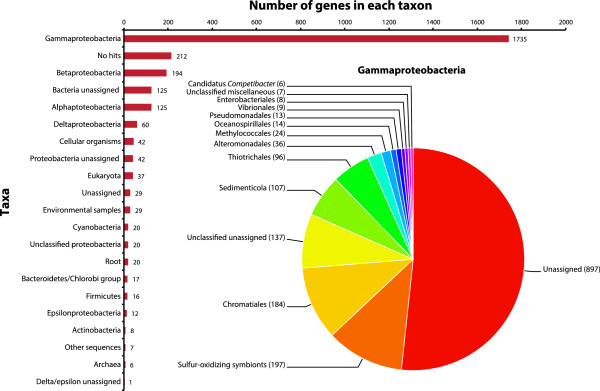
**Taxa assigned to the genes in the** ***S. velum*** **symbiont genome.** The insert chart shows the breakdown of the genes by taxa within the class of γ-proteobacteria (62.9%). The unassigned genes have not been assigned a lower taxon in this analysis. The unclassified genes have not been further classified in the NCBI taxonomy. All the taxa are mutually exclusive.

A model of the symbiont cell based on functional predictions is presented in Figure 1 (see Additional file 3: Table S3 for the list of the corresponding gene products). When grouped into COG categories [37], the largest number of genes within the genome of the *S. velum* symbiont was associated with metabolism of coenzymes, transcription, posttranscriptional modification of proteins, cell division, DNA replication, and energy metabolism (Figure 2). Based on a BLASTN [38] search against the NCBI-nr database analyzed by MEGAN [39], 1,735 of the genes in the genome were assigned to γ-proteobacteria, mainly other sulfur-oxidizing symbionts (197 genes) and bacteria from the order of Chromatiales (184 genes). Among the genes within γ-proteobacteria, 897 could not be assigned to a lower-level taxon in the NCBI taxonomy. 37 genes had the closest matches to eukaryotes and 6 to archaea. No taxa could be assigned to 29 genes, while 212 genes had no hits in the NCBI-nr database (Figure 3). The majority of the sequences designated as “eukaryotic” were hypothetical and produced low percent amino acid identity matches in the BLASTN search.

### Metabolic functions

#### Chemolithotrophy

**Figure 4:**
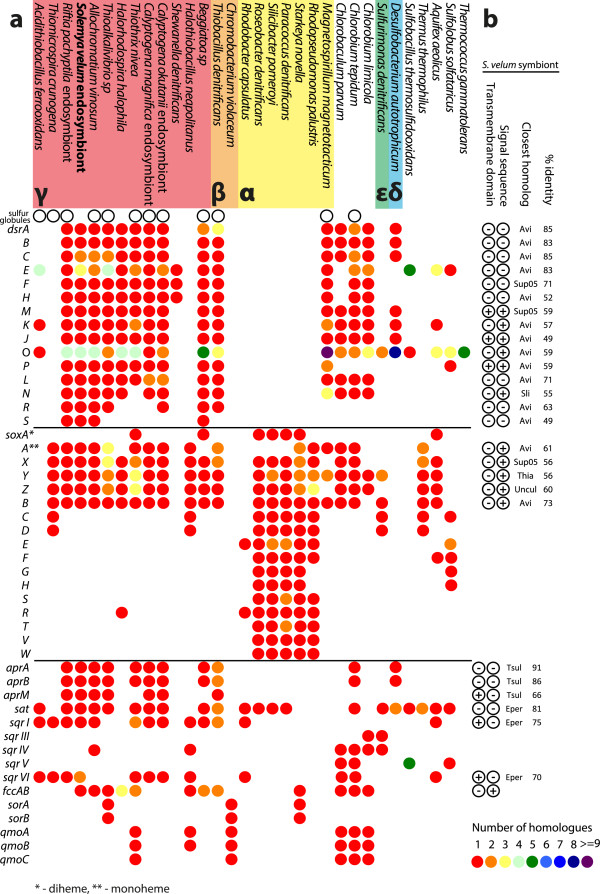
**Comparison of the sulfur oxidation genes between the** ***S. velum*** **symbiont and other SOB. (a)** Presence of genes involved in chemotrophic sulfur oxidation in the symbionts of *S. velum*, other sulfur-oxidizing bacteria and archaea, and sulfate reduction in *D. autotrophicum*, which is included for comparison. Genes encoding pathways for reverse-acting dissimilatory sulfur-oxidation (rDsr) (Drs in *D. autotrophicum*) and periplasmic sulfur-oxidation (Sox), as well as auxiliary proteins, are listed. Numbers of gene homologs in each organism are designated with color. Presence of extra- or intracellular sulfur deposits, i.e., globules, in each organism, as obtained from literature, is indicated with hollow circles. The abbreviations used, the respective full gene product names, and the corresponding NCBI protein ID references in the genome of the *S. velum* symbiont are listed in Additional file 3: Table S3. **(b)** Presence of signal sequences and transmembrane domains in the sulfur-oxidations genes of the *S. velum* symbiont, followed by the list of organisms with the closest known homologs to those genes and their respective BLASTP % identities (Avi - *Allochromatium vinosum*, Sup05 - uncultivated oxygen minimum zone microbe [42], Sli - *Sideroxydans lithotrophicus*, and Thia - *Thiocapsa marina*, Uncul - uncultured organism, Tsul - *Thioalkalivibrio sulfidiphilus*, Eper - *R. pachyptila* endosymbiont).

The *S. velum* symbiont, and chemoautotrophic symbionts in general, are remarkable in their ability to support almost all the metabolic needs of their metazoan hosts with energy derived from thiotrophy. Present genome data illustrate the ability of the *S. velum* symbiont to oxidize both hydrogen sulfide and thiosulfate via diverse pathways, in agreement with previous measurements of symbiont gene expression [40] and *in vitro* experiments showing that both substrates can stimulate carbon fixation in the symbiont [10, 13]. The *S. velum* symbiont genes involved in the oxidation of reduced sulfur species are most closely related to those of the purple sulfur γ-proteobacterium, *Allochromatium vinosum* (Figure 4), in which the genetic components and the biochemical mechanisms of sulfur metabolism have been well characterized [41].

##### Periplasmic sulfide and thiosulfate oxidation

In the periplasm of the *S. velum* symbiont, sulfide, thiosulfate, and, possibly, elemental sulfur, may be oxidized for energy by the Sox system, which is represented in the genome (Figure 4). The encoded SoxYZAXB, flavocytochrome *c* dehydrogenase (FccAB), and type I and IV sulfide-quinone reductases (Sqr) potentially reduce cytochromes *c* and quinones, which along the course of the electron-transport chain translate into membrane-ion gradients, NADH, and ATP, ultimately fueling biosynthetic and other energy-requiring cellular processes, including autotrophy (Figure 1). In *A. vinosum* and the green non-sulfur bacterium, *Chlorobium tepidum*, SoxYZ, SoxAX, and SoxB proteins participate in the formation of transient sulfur deposits as intermediates during sulfur oxidation [43]. In fact, sulfur deposits are common to all known sulfur-oxidizing bacteria (SOB) which, like the *S. velum* symbiont, lack SoxCD sulfur dehydrogenase (Figure 4) [44], including the symbionts of the hydrothermal vent tubeworm, *R. pachyptila*[31, 45], and the clam, *C. magnifica*[22, 46]. Microscopically-detectable intracellular or extracellular sulfur has not been observed either in the symbiont-containing gills of *S. velum* or directly within the symbionts (Cavanaugh, unpublished observation). Absence of sulfur deposits may be attributed to a very rapid consumption of any available reduced sulfur substrate. This agrees with the fact that the *S. velum* symbiont have the highest known carbon fixation rate, and, hence, demand for energy, of all the studied chemosynthetic symbionts, i.e., 65 μmol min^-1^ g of protein^-1^[13] compared to 0.45 μmol min^-1^ g of protein^-1^ of the next highest rate measured in the symbionts of *R. pachyptila*[47]. Alternatively, in the *S. velum* symbiont intermediate sulfur may be stored in a chemical form that is not easily observed microscopically.

##### Cytoplasmic sulfide oxidation

Energy generating oxidation of sulfide to sulfite may be catalyzed in the cytoplasm of the *S. velum* symbiont by the reverse-acting dissimilatory sulfite reductase (rDsr) pathway (Figure 1). All of the enzymes and accessory proteins required for this pathway are encoded in a *dsrABEFHCMKLJOPNRS* operon (Figure 4). While multiple homologues of *dsrC* were identified outside the *dsr* operon, these genes did not encode the two conserved C-terminal cysteines required for the protein to function [48, 49]. The DsrC enzyme likely mediates transfer of electrons from sulfide reductase, DsrAB, to a transmembrane electron transport complex DsrKMJOP, an entry point for electrons derived from cytoplasmic oxidation of sulfur into the electron transport chain [50]. rDsr may be the key energy-generating pathway in the symbiont, as sulfide has a six-fold higher effect on carbon fixation in the *S. velum* symbiosis [13] compared to thiosulfate oxidized by the Sox pathway.

##### Sulfite oxidation

Sulfite generated by rDsr may be further oxidized to sulfate in the cytoplasm by a sequential action of APS reductase (AprABM) and an ATP-generating ATP sulfurylase (Sat) (Figures 1 and 4). Identification of the respective genes agrees with measured Apr and Sat activity in the symbiont-containing *S. velum* tissue [51]. Sulfate generated in this pathway may be exported from the cytoplasm via a sulfate-bicarbonate antiporter SulP (Figure 1). While electrons obtained from the oxidation of sulfide, thiosulfate, and, possibly, elemental sulfur by Sox and rDsr are shuttled into the electron transport chain, energy obtained from the oxidation of sulfite is immediately available in the form of ATP.

#### Bioenergetics

The *S. velum* symbiont is thought to harvest energy from reduced sulfur oxidation with oxygen. Interestingly, its genome also encodes other respiratory pathways suggestive of diverse metabolic strategies. Based on the gene content, the symbiont may utilize multiple electron donors such as hydrogen, pyruvate, malate, succinate, and formate, and use alternative electron acceptors such as nitrate and dimethyl sulfoxide (DMSO). Furthermore, unlike any chemosynthetic symbiont studied to date, the *S. velum* symbiont contains genes that may allow it to preferentially establish H^+^ and Na^+^ electrochemical membrane gradients during each step of respiration and to selectively utilize them for ATP synthesis, solute transport, and pH control. This high degree of respiratory flexibility encoded in the *S. velum* symbiont genome suggests that this bacterium is adapted to a highly variable environment.

##### Rnf complexes

The versatile electron transport chain of the *S. velum* symbiont may utilize electron donors like ferrodoxins, which have a redox potential as negative as -500 mV [52], compared, for example, to -400 mV of S_2_O_3_^2-^ and -270 mV of H_2_S. The reversible oxidation of ferrodoxins coupled to the reduction of NAD^+^ in the *S. velum* symbiont may be catalyzed by the H^+^ or/and Na^+^-motive Rnf complexes (Figure 1) encoded in the genome by two complete *rnfABCDGE* (*rnf1*) and *rnfBCDGEA* (*rnf2*) operons. The organization of these genes in the operons is conserved with other bacteria, suggesting that these clusters did not arise from duplication. Previously, only *Axotobacter vinelandii* and *Desulfobacterium autotrophicum* were known to harbor two *rnf* operons [52]*.* Based on the presence of genes for pyruvate:ferredoxin oxidoreductase located between *rnfB2* and *rnfC2*, pyruvate may serve as an electron donor for at least one of the Rnf complexes. In general, *rnf* genes are distributed mainly among obligate and facultative anaerobes, including many pathogens that colonize oxygen-limited host tissues [52]. Together with the fact that ferrodoxins play a key role in anaerobic metabolism [53], this suggests that the *S. velum* symbiont, as well as other sequenced chemosynthetic symbionts, which all contain *rnf* genes, may be capable of facultative anaerobiosis.

##### Hydrogenases

Hydrogen is another highly electron negative reductant (-420 mV) that the *S. velum* symbiont may harness for the reduction of the quinone and the NAD^+^ cellular pools (Figure 1). Hydrogen oxidation is suggested by the presence in the symbiont genome of *hup* and *hox2* operons encoding an uptake and a bidirectional hydrogenase, respectively. The two subunits of the symbiont [Ni-Fe]-uptake hydrogenase, HupSL, are most similar in amino acid sequence to HupS and HupL proteins from the symbionts of the tubeworms, *R. pachyptila* and the *T. jerichona*, (73% and 78% identity for the S and L subunits respectively), the sulfur bacterium, *Thiocapsa roseopersicina,* (68 and 74%), and the symbionts of the scaly-foot snail, *C. squamiferum*, (50 and 53%). In *T. roseopersicina,* HupSL has been experimentally demonstrated to reduce quinones of the respiratory chain with H_2_[54, 55]. Unlike all the other γ-proteobacteria containing HupSL, the *hup* operon in the *S. velum* symbiont does not encode the di-heme cytochrome *b*, which is necessary to link H_2_ oxidation to quinone reduction in the cellular membrane [56]. However, a [Ni-Fe] hydrogenase cytochrome *b* homolog was found on a different genomic scaffold. Though this discordant gene organization is unlike that in other H_2_ oxidizers, it is possible that the identified cytochrome *b* may act in tandem with HupSL to enable H_2_ oxidation.

Apart from potentially reducing the respiratory quinone pool with H_2_, the symbiont, by means of a bidirectional hydrogenase, may produce H_2_ by oxidizing NAD^+^. The *S. velum* symbiont Hox2FUYH enzyme complex is most similar in amino acid sequence (63-66%) to the recently-characterized NAD^+^-reducing [Ni-Fe]-hydrogenase from *T. roseopersicina*, which can operate in reverse, generating H_2_ when the high reduction state of the dinucleotide pool is growth-limiting [57]. As H_2_ concentrations available to the *S. velum* symbiont have not been measured, it is unknown whether the H_2_ oxidation contributes to primary production to the degree that has been recently demonstrated in a hydrothermal vent symbiosis [58].

##### Primary ion pumps

NADH (-320 mV), potentially derived from oxidation of H_2_ or heterotrophic metabolism (see Heterotrophy) in the *S. velum* symbiont, could be converted into an electrochemical gradient by two NADH:quinone oxidoreductases. The genome of the symbiont encodes the conventional H^+^-translocating quinone-reducing NADH dehydrogenase (NdhABCDEFGHIJKLMN), a homolog of the mitochondrial Complex I, as well as an alternative Na^+^-translocating NADH dehydrogenase (NqrABCDEF) (Figure 1). While Complex I is ubiquitous in bacteria, Nqr is found mainly in pathogenic and marine species [59]. Among symbiotic bacteria, *nqr* genes have so far been described only in *Buchnera* spp., an obligate endosymbiont of aphids [60]. The *S. velum* symbiont may be able to switch between Complex I and Nqr, preferentially generating either H^+^ or Na^+^ electrochemical gradients. Thus, depending on the cellular requirements, the symbiont may synthesize ATP (see ATP synthases) and regulate pH (see Ion gradient driven transporters) independently from each other.

##### Quinone reductases

Apart from the electron donors such as sulfur and NADH, the *S. velum* symbiont may be able to directly reduce its quinone pool with a number of other substrates. This is suggested by the presence of genes encoding malate:quinone oxidoreductase (Mqo), succinate dehydrogenase (ShdCDAB), homologous to Complex II in mitochondria, and formate dehydrogenase-O (FdoGHI) (Figure 1). This is the first report of FdoGHI in a chemosynthetic symbiont genome. In *E. coli* this enzyme, which is common to facultative anaerobes [61], is used in formate-dependent oxygen respiration, allowing the bacteria to rapidly adapt to shifts from aerobiosis to anaerobiosis [62]. The presence of FdoGHI is additional evidence that the *S. velum* symbiont may be capable of facultative anaerobiosis (see Rnf complexes).

The genome-encoded quinol-cytochrome-*c* oxidoreductase (*bc*_*1*_, Complex III homologue) potentially links oxidation of quinols to the generation of a proton membrane gradient and the reduction of terminal electron acceptors (Figure 1), discussed next.

##### Terminal oxygen reductases

Similar to most aerobic and microaerophilic bacteria*,* the genome of the *S. velum* symbiont encodes three types of H^+^-motive terminal oxygen reductases (Figure 1), which suggest a capacity to respire O_2_ over a wide range of concentrations. The genome contains a *ccoNOQP* operon encoding a *cbb*_*3*_ cytochrome oxidase, which is known to function at nanomolar O_2_ concentrations in the nitrogen-fixing plant symbionts, *Bradyrhizobium japonicum*[63], and in the microaerophilic human pathogens, *Campylobacter jejuni, Helicobacter pylori*, and *Neisseria meningitidis*[64]. The genome also encodes a *aa*_*3*_ cytochrome oxidase (CoxAB), which is thought to function primarily under atmospheric oxygen concentrations [65] and is the only terminal oxidase in the symbionts of the bivalves *C. magnifica*[22] and *C. okutanii*[20]. The third terminal oxidase identified in the symbiont genome is a *cydAB*-encoded quinol oxidase, which is thought to oxidize quinols instead of cytochromes. CydAB may operate when an excess of reductants, potentially coming from the host, limits metabolic turnover and a redox balance needs to be achieved [66]. The observed diversity of terminal oxygen reductases indicates that the supply of oxygen to the symbionts may fluctuate over time or between free-living and symbiotic stages, necessitating adjustments in respiratory metabolism.

##### Alternative terminal reductases

When oxygen is limited or unavailable, potentially either through competition for oxygen with the host or if the symbionts find themselves in the anoxic sediment that surrounds the burrow, the *S. velum* symbiont may be capable of using terminal electron acceptors other than oxygen. Although it is unknown whether the symbiont-containing gill bacteriocytes ever become anaerobic, the presence of genes for periplasmic NO_3_^-^ reductase (*napFDAGHBC*) suggests that symbiont energy generation may involve electron transfer to nitrate, which is available in the porewater surrounding *S. velum* at concentrations of ~1-10 μM ( [67], in preparation). The structure of the symbiont *napFDAGHBC* operon is consistent with that of enteric bacteria that are thought to use Nap for effectively scavenging nitrate during anaerobic growth under nitrate-limited conditions (5 μM) [68]. The symbiont genome also encodes a DMSO reductase (*dmsABC*), which suggests an ability to respire dimethyl sulfoxide (DMSO), a breakdown product of dimethylsulfoniopropionate (DMSP) produced, for example, by marine algae. DMSO is available at nanomolar concentrations in the coastal eutrophic environments inhabited by *S. velum*[69], and Dms genes are common to many marine sediment-dwelling bacteria, e.g., *Beggiatoa* and *Shewanella*[70, 71].

##### ATP synthases

Based on the genome data, both H^+^ and Na^+^ membrane gradients, established along the course of the electron transport chain during respiration, may drive ATP synthesis in the *S. velum* symbiont via either H^+^- or Na^+^-dependent ATP synthases (Figure 1). The H^+^-specificity of the F_0_F_1_-type ATP synthase is suggested by the presence of two characteristic transmembrane helixes within the *c* subunit. In contrast, an A_0_A_1_-type ATP synthase detected in the genome contains the characteristic Na^+^-binding PXXXQ motif I and ES motif II in the rotor subunit K. While proton-translocating ATP synthases are predominant in bacteria, Na^+^-coupled ATP synthesis driven by respiration has recently been recognized in some marine and pathogenic species [72, 73]. To our knowledge, this is the first report of a Na^+^-translocating ATP synthase in a chemosynthetic symbiont.

##### Ion gradient driven transporters

Cellular roles of the H^+^ and Na^+^ gradients in the *S. velum* symbiont appear to extend beyond ATP synthesis. Besides ATP synthases, the genome encodes diverse Na^+^:substrate symporters and numerous Na^+^:H^+^ antiporters, including the multi-subunit MrpEFGBBCDD complex (Figure 1). These transporters, together with ATP synthases and respiratory ion pumps, may establish and consume simultaneous transmembrane gradients of protons and sodium ions in the symbiont [72]. These parallel cycles of H^+^ and Na^+^ would allow the *S. velum* symbiont to synthesize ATP and maintain pH homeostasis via two separate mechanisms.

#### Carbon metabolism

Autotrophic carbon fixation, fueled chiefly by sulfur oxidation, is the principal process in the *S. velum* symbiont, supplying both the symbiont and the host with organic carbon [14]. While previous studies focused primarily on RuBisCO [10, 74], the key enzyme of the Calvin cycle for CO_2_ fixation and the most highly expressed gene in the symbiont [40], our current analysis identified genes that encode catalytic components required for CO_2_ fixation and storage, including the pyrophosphate-dependent phosphofructokinase, which has been hypothesized to command a more energy efficient variant of the cycle [22, 75–77]. Furthermore, the genome of the *S. velum* symbiont contains the gene for α-ketoglutarate dehydrogenase – the key enzyme of the tricarboxylic acid cycle (TCA), suggesting that the symbiont can respire organic carbon and may not be obligately autotrophic.

##### Autotrophy

The genome of the *S. velum* symbiont encodes a version of the Calvin cycle which appears to be prevalent in chemosynthetic symbionts but may also operate in a few free-living bacteria [75–77]. In these organisms genes for fructose 1,6-bisphosphatase and sedoheptulose 1,7-bisphosphatase, which process obligate intermediates in the cycle, are absent. Instead, the role of the missing enzymes may be performed by a single reversible pyrophosphate-dependent phosphofructokinase (PPi-PFK), the gene for which was identified in the genome of the *S. velum* symbiont (Figure 1). The ability of this enzyme to dephosphorylate fructose 1,6-bisphosphate and sedoheptulose 1,7-bisphosphate *in vitro* was demonstrated for the PPi-PFK from *Methylococcus capsulatus*[75], which shares 73% amino acid sequence identity with the homologue from the *S. velum* symbiont. Notably, during dephosphorylation this enzyme generates pyrophosphate, which bears a high-energy phosphate bond unlike the orthophosphate liberated by fructose 1,6-bisphosphatase and sedoheptulose 1,7-bisphosphatase. In *M. capsulatus*[75] and in the chemosynthetic symbionts of *R. pachyptila*[76] and the oligochete, *O. algarvensis*[77], it was proposed that the pyrophosphate produced this way could be converted into a proton gradient by a membrane-bound proton-pumping pyrophosphatase (V-type H^+^-PPase) co-encoded with the PPi-PFK. This proton gradient could then be used for ATP synthesis. Compared to the classical Calvin cycle [78], this mechanism may allow bacteria to spend up to 9.25% less energy on CO_2_ fixation [77]. Judging from the similar gene content, this version of the cycle may also be at work in the symbionts of the vent clams, *C. magnifica*[22] and *C. okutanii*[20]. Apart from the membrane-bound V-type H^+^-PPase, the *S. velum* symbiont genome also encodes a soluble pyrophosphatase (PPase) immediately upstream of the PPi-PFK gene. The PPase cannot convert the energy of pyrophosphate into a proton gradient but, by controlling the availability of pyrophosphate, may serve to regulate the catalytic direction of the PPi-PFK, which may also participate in glycolysis as a kinase. This additional PPase suggests that it may be important for the *S. velum* symbiont to control the direction of its carbon flux to a higher degree than what has been seen in other chemosynthetic symbionts.

##### Carbon Flux

Carbon fixed by the *S. velum* symbiont may be stored as polyglucose or fed into catabolic and anabolic reactions (Figure 1). The overall direction of the metabolic carbon flux in the symbiont can be controlled by at least two putative mechanisms. First, the reversible PPi-PFK, participating in the Calvin cycle as discussed above, may also phosphorylate fructose 6-phosphate during glycolysis. PPi-PFK appears to be the only enzyme encoded in the genome that could catalyze both the forward and the reverse reactions. The directionality of the catalysis may depend on the concentration of pyrophosphate and the other substrates of the enzyme in the cytoplasm [79], since this PPi-PFK is likely nonallosteric [75]. Second, the two encoded glyceraldehyde 3-phosphate dehydrogenases, GapA and GapB, may be specific to glycolysis and the Calvin cycle/gluconeogenesis, respectively, by analogy to the homologous enzymes in *Staphylococcus aureus*[80]. In the symbiont genome, *gapB* is adjacent to the gene for transketolase, an enzyme in the Calvin cycle, further suggesting that these two Gap proteins may play a role in regulating the direction of the carbon flux either in the direction of glycolysis or the Calvin cycle and gluconeogenesis. The symbionts of *C. magnifica, C. okutanii*, *R. pachyptila, T. jerichona*, and the scaly snail possess just a single *gap* gene, which has a much higher amino acid sequence identity to *gapB* than to *gapA* from the *S. velum* symbiont*.* In line of the above evidence the symbiont of *S. velum* appears to be distinct from other chemosynthetic symbionts in placing a stronger emphasis on controlling the direction of its carbon flux.

##### Heterotrophy

The *S. velum* symbiont is the third chemosynthetic symbiont, along with the γ3-symbiont of *O. algarvensis*[34] and the intracellular γ-proteobacterial symbionts of the scaly-foot snail [33], known to encode all of the enzymes required for the complete TCA cycle, and, therefore, could oxidize organic carbon for energy (Figure 1). All of the other sequenced chemosynthetic symbionts lack genes for α-ketoglutarate dehydrogenase and citrate synthase, which suggests their obligate autotrophy [81].

Furthermore, genes for the glyoxylate bypass of the TCA cycle, encoding isocitrate lyase and malate synthase, were also found in the genome of the *S. velum* symbiont (Figure 1). These enzymes could allow the symbiont to grow on various carbon sources, including acetate and other two-carbon compounds, [82] or rapidly replenish intermediates of biosynthetic reactions. The presence of the glyoxylate bypass and the TCA cycle suggests that the symbiont may be a facultative mixo- or heterotroph. The adaptive role of having both heterotrophic pathways, however, is unclear, and may relate either to the intracellular conditions specific to this particular symbiosis or to the yet unconfirmed host-free existence of the symbiont.

#### Nitrogen metabolism

Ammonia, abundant in the sediment where *S. velum* burrows, is the main form of nitrogen assimilated by the symbiosis [83]. It has been suggested that the symbionts incorporate ammonia into biomass, which is then transferred to the host ( [67] in preparation), a process which has been described for the chemosynthetic symbionts of the hydrothermal vent tubeworm *Ridgeia piscesae*[84]. The presence of assimilatory nitrogen pathways in the *S. velum* symbiont genome corroborate this hypothesis.

##### Nitrogen assimilation

Extracellular ammonia may be imported by the symbiont via specific AmtB transporters and incorporated into glutamate and glutamine, which serve as amino group donors for the other nitrogen-containing compounds in the cell (Figure 1). The *S. velum* symbiosis comes in contact with 20–100 μM concentration of ammonia in its coastal environment ( [67] in preparation). Thus, it is not surprising that, unlike the chemosynthetic symbionts found at nitrate-rich (40 μM) hydrothermal vents [85, 86], the *S. velum* symbiont lacks *nar* genes for nitrate reductases capable of assimilatory nitrate reduction [32, 87–89]. Assimilation of ammonia has been previously demonstrated in the gills of *S. velum*, but was initially ascribed to the activity of the host glutamine synthetase (GS) [88]. The present analysis identified *glnA*, the gene that encodes GS, in the genome of the symbiont. A preliminary transcriptional study showed *glnA* to be one of the fifty most highly transcribed genes in the symbiont [40]*.* The biosynthetic pathways reconstructed on the basis of gene content suggest that the symbiont has the ability to make all of the 20 proteinogenic amino acids. The amino acid prototrophy of the symbiont is in keeping with its proposed role in providing most, if not all, of the host’s nutrition [14, 15].

##### Urea metabolism

Host urea may serve as an additional source of assimilatory nitrogen for the *S. velum* symbiont. The identified *ureHABCEFG* operon encodes a cytoplasmic urease UreABC, which can hydrolyze urea, releasing ammonia that may be re-utilized by the symbiont. Urea can enter the bacterial cell by passive diffusion [90], but under nitrogen starvation the symbiont may be able to take it up more rapidly via an ABC-transporter UrtABCDE, encoded directly upstream of the *ure* genes. Among chemosynthetic symbionts, urease genes have been previously described only in the γ-symbionts from the marine oligochaete worm *O. algarvensis*[34, 77], which, like *S. velum*, lives in coastal sediments. The sequenced chemosynthetic symbionts from hydrothermal vents lack urease genes, even though some of their host organisms, for instance *R. pachyptila*[91], are known to produce urea. This discrepancy may be accounted for by the fact that in coastal sediments urea is also present outside the host in the pore water ( [67] in preparation).

##### Taurine synthesis

The *S. velum* symbiont may also provide its host with nitrogenous osmoregulants, such as the non-proteinogenic amino acid taurine [92]. In the host tissues, taurine accounts for up to 70% of the total free amino acids and shows an isotopic composition (δ^13^C, δ^14^N, δ^34^S) suggestive of symbiont origin [93]. Synthesis of taurine may be accomplished by the two homologs of the reversible taurine dioxygenase (TauD) encoded in the symbiont genome. Taurine could be actively secreted to the host by the TauABCD ABC transporter, the genes for which were found to contain a conserved binding domain for sulfonate, characteristic of the taurine molecule. Since taurine synthesis requires sulfite [94], one of the final intermediates in sulfur oxidation, this pathway could serve to dispose of SO_3_^2-^, and, thus, to drive forward sulfur oxidation in the *S. velum* symbiont, benefiting both the host and the symbiont.

### Membrane-associated functions

The diversity of membrane-associated functions encoded in the genome of the *S. velum* symbiont suggests that the symbiont is fully autonomous of its host in this aspect of its physiology. Other bacteria, which, like the symbiont*,* are thought to be obligately intracellular [17], have lost genes required for the production of a cellular envelope, transport of solutes across the plasma membrane, sensing of the extracellular environment, as well as motility. These bacteria instead rely on their hosts to perform these functions or no longer require them.

#### Production of cellular envelope

The *S. velum* symbiont appears capable of synthesizing and assembling a cytoplasmic membrane, a peptidoglycan layer (PG), and an outer membrane populated by lipopolysaccharides (LPS), which constitute a cellular envelope. While these abilities are typical of the free-living γ-proteobacteria, two aspects in particular stand out in the context of the symbiotic life-style*.* First, given the identified genes for the biosynthesis of fatty-acids, the symbiont may build components of its plasma membrane mostly from *cis*-vaccenic acid (18: lω7) (Figure 1). According to a previous analysis of lipid composition in *S. velum*[95], this unsaturated fatty acid and its derivatives are the main constituents of cellular membranes in the symbiont and the host alike. Furthermore, the isotopic signature of the host’s lipids indicates that they are bacterial in origin [95]. Second, the identified genes for the synthesis of lipopolysaccharides (Figure 1) suggest that the symbiont may be able to assemble the LPS structures that are known to be sufficient for growth of *E. coli*[96]. Most intracellular symbionts that live within a host derived membrane, like the *S. velum* symbiont [10]*,* lack LPS biosynthetic genes and are unable to replicate on their own [97]. However, the symbionts which have the genes to synthesize LPS tend to either live directly in the cytoplasm [97] and have to make their own cellular envelope or, like the symbionts of *R. pachyptila*[98], exist extracellularly for part of their life. Therefore, the symbiont of *S. velum* may not only be able to make a fully functional cellular envelope and supply some of its components to its host, but may also be capable of living outside the bacteriocytes.

#### Membrane transport

##### Transporters

The number of transporters encoded in the genome of the *S. velum* symbiont exceeds what has been found in other intracellular bacteria (Table 2). The diversity of genes for solute transport (Figure 1) suggests that the symbiont has an extensive chemical communication with their environment. The *S. velum* symbiont may use these transporters to import metabolic substrates and enzyme cofactors and export products of its biosynthesis to sustain the physiology of the host. It is known that fixed organic carbon is transferred from the symbiont to the host within minutes [99], which suggests a transport mechanism, since direct digestion of symbionts by host cells would likely take hours to days [100]. Such transport could be accomplished by exporters of amino acids (EamA), carboxylates (CitT), and fatty acids (FadLD), all of which are encoded in the genome. Moreover, some of the importers found in the genome may also act as exporters, depending on the cellular environment [101]. Thus, the *S. velum* symbiont maintains a repertoire of transporters that may negotiate diverse chemical exchanges with the environment and, on the other hand, allow it to provide nutrients to the host without being digested.

**Table 2:** **Comparison of extracellular transport genes in the** ***S. velum*** **symbiont, other symbiotic and free-living bacteria**

Organism	Lifestyle	Transporter gene ratio to ***S. velum*** endosymbiont	Genome size (Mb)	Total number of genes involved in transport	Transporter genes per Mb of genome	ATP-dependent transporters	Secondary transporters	Phosphotransferase systems	Ion channels	Unclassified transporters	Protein secretion systems	Outer membrane transporters
***Solemya velum*** **endosymbiont**	**Intracellular symbiont**	**1.00**	**2.7**	**224**	**75.2**	**100**	**70**	**1**	**5**	**5**	**17**	**26**
*C. magnifica* endosymbiont	OIS*	0.14	1.16	32	27.6	18	6	0	3	1	0	4
*C. okutanii* endosymbiont	OIS	0.15	1.02	34	33.3	16	10	0	2	3	0	3
*Buchnera aphidicola* APS	OIS	0.07	0.64	16	25.0	5	3	5	1	0	0	2
*Sulcia muelleri* GWSS	OIS	0.03	0.25	7	28.0	4	2	0	0	0	0	1
Ca. *Blochmannia floridanus*	OIS	0.12	0.71	27	38.0	7	12	3	2	0	0	3
*Wigglesworthia glossinidia*	OIS	0.11	0.70	25	35.7	9	14	0	2	0	0	0
*Baumannia cicadellinicola*	OIS	0.13	0.69	28	40.6	11	10	3	1	0	0	3
*R. leguminosarum* bv. Viciae 3841	FIS*	2.47	7.75	553	71.4	281	203	7	18	2	13	29
*Frankia alni* ACN14a	FIS	1.05	7.50	236	31.5	114	106	0	12	1	1	4
*Vibrio fischeri* MJ11	Extracellular symbiont	1.80	4.50	404	89.8	138	141	12	10	6	46	51
*Wolbachia pipientis* wSim	OIS/parasite	0.21	1.06	48	45.3	19	28	0	0	1	8	0
*Rickettsia prowazekii* MadridE	Intracellular parasite	0.21	1.10	48	43.6	15	30	0	1	1	0	5
*Escherichia coli* K-12-MG1655	Commensal	1.58	4.64	354	76.3	74	235	29	13	2	3	35
*Klebsiella pneumoniae* kp342	Commensal	2.82	5.92	632	106.8	160	336	44	17	4	37	34
*Thiomicrospira crunogena* XCL-2	Free-living sulfide oxidizer	0.73	2.43	163	67.1	38	58	0	10	3	35	19
*Allochromatium vinosum* DSM 180	Free-living sulfide oxidizer	0.89	3.67	199	54.2	81	52	5	8	7	14	32
*Sulfurimonas denitrificans* DSM 1251	Free-living sulfide oxidizer	0.43	2.20	97	44.1	32	52	0	10	3	6	25
*Methylococcus capsulatus* Bath	Free-living methanotroph	0.76	3.30	171	51.8	56	60	0	6	2	16	31
*Thermodesulfovibrio yellowstonii* DSM 11347	Free-living sulfate reducer	0.39	2.00	88	44.0	31	34	0	3	2	3	15

OIS - obligate intracellular symbiont; FIS - free-living intracellular symbiont.

##### Multi-drug efflux pumps

The *S. velum* symbiont genome contains at least five sets of genes encoding multi-drug efflux pumps (AcrAB-TolC), suggesting the ability to expel host-derived antimicrobial agents. A comparable genetic capacity for the AcrAB-TolC efflux system has been found in bacteria, such as the plant symbiont *Rhizobium leguminosarum,* that have a free-living stage, but not in bacteria that are obligately intracellular (Table 2, ATP-dependent transporters). The plant host of *R. leguminosarum* manipulates the cellular fate of its symbionts using antimicrobial-like peptide factors [102]. As a result, *R. leguminosarum* undergoes cell elongation and genome replication but looses its ability to divide. Only a small number of *R. leguminosarum* cells remain vegetative [103]. A very similar morphological differentiation of the symbiont has been observed in *S. velum*[104]. Assuming the bivalve host also uses peptide factors to control its symbiont, the *S. velum* symbiont may rely on the efflux pumps to maintain a small undifferentiated population in the bacteriocytes for transmission to future host generations.

#### Sensory mechanisms and motility

The *S. velum* symbiont appears well equipped to sense extracellular chemical changes, consistent with its inferred ability to maintain a complex chemical exchange with the environment. Over forty transmembrane chemoreceptors are encoded in the genome of the symbiont. Almost half of them have one or more conserved PAS domains and therefore may play a role in sensing oxygen levels and redox potentials. To relay sensory information, the majority of the receptors contain either a diguanylate cyclase (GGDEF) or a histidine kinase (HisKA) signaling domain. Movement and surface attachment using type IV pili, known as twitching motility, are the processes that may be regulated by chemosensory signal transduction in the *S. velum* symbiont (Figure 1)*.* For example, in the genome of the symbiont a chimeric gene containing PAS, GGDEF, and cyclic-diguanylate receptor (EAL) domains is co-located with *pilEY1XWVT* genes required to assemble a functional pilus. Furthermore, the symbiont genome contains *pilGIJ-cheAW* genes, which encode a transmembrane chemotaxis sensor protein, HisKA, and a DNA-binding response regulator, and are known to control twitching motility in other bacteria [105]. The symbiont may use the contractile pili to direct its movement in the environment with regard to chemicals gradients, and, potentially, also rely on the same mechanism to find and colonize new hosts.

### Mobile genetic elements

The *S. velum* symbiont genome contains two major types of mobile elements, integrative and conjugative elements (ICEs) and insertion sequences (IS). The genome contains 25 insertions from 12 different ICE families (Table 3) as well as 53 copies of four different IS elements (Table 4). In total, these elements comprise 2.6% of the genome. No gene interruptions were associated with these elements. This large number and diversity of mobile elements suggest that this bacterium may come into contact with other bacterial lineages more often than expected for most vertically transmitted intracellular symbionts. Indeed, the abundance of mobile genetic elements in bacterial genomes has been shown to correlate with ecological niche. While there is considerable overlap between the amounts of mobile elements hosted by free-living and facultative intracellular bacteria, obligate intracellular bacteria that undergo faithful vertical transmission consistently have few or no mobile elements [106].

**Table 3:** **ICE mobile genetic elements in the** ***S. velum*** **symbiont genome**

ICE element	Copies	Length, bp
ICEVchLao1	1	834
ICEVchBan7	1	432
ICEVchBan9	2	429, 888
ICEVchInd5	1	282
ICEVchMex1	1	561
ICEVflind1	2	405, 729
ICEPalBan1	1	1389
ICEPdaSpa1	5	300, 387, 622, 939, 3568
ICESpuPO1	3	549, 627, 648
ICEPmiUSA1	1	1290

**Table 4:** **Insertion sequence mobile genetic elements in the** ***S. velum*** **symbiont genome**

Family/Element	Copies	Length, bp	Terminal inverted repeats
IS30	30	1071	ATTCAA
IS3/IS407	18	1219	CCCCCA/CCCCCAA(C/T)AAGT
IS30	1	900	CAACCGTTTCAAT
IS5/IS5	1	1638	ACCCAAGGTA
IS481	1	1271	GAGACATCATTTACA
IS30	1	1137	TGATGTACGGGTCCGA
Unknown	1	1848	CCCCTTCG

Two hypothesized life and evolutionary history scenarios may explain the observed mobile element content in the *S. velum* symbiont. One of them is a relatively recent shift to intracellularity, resulting in an expansion of mobile elements [107, 108]. Alternatively, the symbionts may undergo regular or occasional horizontal transmissions between hosts and at that time encounter opportunities for recombination between strains. For example, sporadic episodes of horizontal transmission in the primarily maternally transmitted insect symbiont, *Wolbachia*, have resulted in the acquisition and maintenance of novel mobile elements [109, 110]. In fact, horizontal transmission or host-switching has likely occurred in the history of symbionts of bivalves [111] including members of the genus *Solemya*, as 16S rRNA phylogenetic analyses show that these symbionts do not comprise a monophyletic clade [5, 11]. Additionally, many of the genes in the *S. velum* symbiont genome are most closely related to disparate bacterial taxa (Figure 3), suggesting that horizontal gene transfer may have occurred in the past. These preliminary lines of evidence support the hypothesis that horizontal symbiont transmission has occurred. However, more information is needed about the distribution and relationships of the mobile elements among intra-host and inter-host *S. velum* symbiont populations before these hypotheses can be differentiated.

## Conclusions

Many of the features commonly encoded in the genomes of chemosynthetic symbionts were observed in the genome of the *S. velum* symbiont alongside an array of genes unique to this bacterium. Potential adaptations to the symbiotic lifestyle, such as a more energy-efficient version of the Calvin cycle, were shared with the other sequenced chemosynthetic symbionts. The genes that set the *S. velum* symbiont apart from the others were those that encoded the TCA and the glyoxylate cycles, DMSO and urea reductases, as well as the highly branched electron transport chain. These functions may relate to the fact that the *S. velum* symbiosis lives in eutrophic sediment, unlike the oligotrophic environments inhabited by other chemosynthetic symbioses, e.g., those of *R. pachyptila, C. magnifica,* and *O. algarvensis*.

The *S. velum* symbiont has long been considered to be vertically transmitted [17], but our genomic analyses are inconsistent with predictions based on other vertically transmitted obligately-intracellular bacteria. The *S. velum* symbiont’s genetic repertoire is replete with genes for chemosynthesis, heterotrophy, bioenergetics, nitrogen metabolism, cell maintenance, motility, communication, and exchange with the environment. Thus, with regard to the functional gene content, but also the genome size and GC composition, the genome is more similar to those of free-living sulfur-oxidizing bacteria (Table 1). Furthermore, the genome contains mobile elements that are comparable in numbers reported for horizontally-transmitted obligately-intracellular bacteria. These divergent lines of evidence suggest that the evolutionary life history of the *S. velum* symbiont may be more complicated than previously hypothesized. This could include, but may not be limited to, an opportunistic generalist lifestyle, a facultative symbiosis with a mixed transmission mode, or a very recent obligate association with the host for this clade of bacteria potentially on a path to a new type of a cellular organelle.

## Methods

### Specimen collection and DNA preparation

*S. velum* individuals were collected by the staff of the Marine Resource Center of the Marine Biological Laboratory (MBL), Woods Hole, MA from reducing sediment of shallow eelgrass beds near Naushon Island, Woods Hole, MA in 2006, 2007, and 2012. The collection was performed in accordance with state collecting permit issued by the Division of Marine Fisheries and in compliance with all local, regional and federal regulations, including the Convention on Biological Diversity and the Convention on the Trade in Endangered Species of Wild Fauna and Flora. The excised gills were macerated in the laboratory using a dounce homogenizer in 5 ml of 0.2 μm filtered seawater (FSW) per bivalve. Homogenates were passed through 100 μm and 5 μm nylon filters (Small Parts Inc. #CMN-0105-A and CMN-0005-A) and centrifuged at 5,000 × g for 5 minutes at 4°C. The pellet was resuspended in FSW, pelleted, and resuspended in 1x TAE buffer. 50 g molten 2% agarose (SeaKem® #50152) in 1x TAE was added to make plugs for genomic DNA extraction. The hardened plugs were treated with DNAse I (0.25U/50 μl) at 37°C for 10 minutes and then equilibrated in TE buffer for 30 minutes at room temperature. Agarose plugs were further processed using CHEF Mammalian Genomic DNA Plug Kit from Bio-Rad Laboratories (#170-3591) according to the manufacturer’s instructions. The protocol for pulse field gel electrophoresis (PFGE) and isolation of the bacterial chromosomes from the agarose plugs was adapted from Gil [112].

### Genome sequencing and assembly

Genomic bacterial DNA was sequenced at the Institute for Genomic Research (TIGR), the Joint Genome Institute (JGI), and the University of California, Davis, using a diversity of sequencing technologies. Two Sanger libraries of 3–4 Kb and 10–12 Kb insert sizes were constructed as previously described [113]. Sequencing of these Sanger libraries resulted in 110,187 reads with N50 of 969 bp and the average coverage depth of 8x. Subsequently, using Roche 454 technology, 387,143 sequencing reads with the N50 of 207 bp and the average coverage depth of 13x were obtained. Then, 25,635,107 Illumina sequencing reads were generated. The Illumina sequences were 35 bp long and had the average coverage depth of 150x. These Sanger, Roche 454, and Illumina sequences were assembled using the Paracel Genome Assembler (Paracel Inc., Pasadena, CA) into 68 contigs. Next, symbiont DNA was sequenced using Pacific Biosciences (PacBio) technology, resulting in 150,000 reads with N50 of 4,966 bp and 9x coverage depth. The insertion and deletion (indels) errors, typical of the PacBio data [114], were reduced from 4% to 0.2% with Illumina paired-end sequences (500x coverage) using PacBioToCA program [115] available as a part of SMRT Analysis software package version 1.4 distributed by the Pacific Biosciences [116]. The error correction step also removed any PacBio sequences of the host origin, which, given the abundance of the symbionts in the gill tissue, had Illumina coverage below 5x. The Illumina data used for the error correction were generated as part of a different study and came from a specimen obtained at a different location (Point Judith, RI) than the rest of the genomic data. Due to the extent of the intra-species genomic sequence variation across geographic localities (Russell et al., in preparation), these Illumina data could not be used to supplement the genome assembly but were sufficient to correct the majority of sequencing indel errors in the PacBio reads. The error-corrected 54,684 PacBio sequences with N50 of 1,409 bp were used to connect the previous 68 genomic contigs into 30 larger scaffolds using the Automated Hybrid Assembly (AHA) module of SMRT Analysis. The resulting 7 gaps within the scaffolds, 2,272 bp in total, were then filled in with the PacBio error-corrected sequences using the PBJelly software tool [117], reducing the number of gaps to 4 and the total gap length to 100 bp. After discarding 20 of the smallest low coverage (2-9x) scaffolds that contained mostly eukaryotic genes (>65%), identified as described below, only 10 scaffolds were retained as a part of the symbiont genome.

### Sequence analysis

Open reading frames (ORFs) on *S. velum* symbiont scaffolds were predicted using Glimmer [118], Prodigal [119], and GeneMarkS [120]. The software parameters used to perform these analyses are listed in Additional file 4: Table S4. Once identified, the ORFs were translated into protein-coding sequences and queried against the UniProt Reference Clusters (UniRef90) (20 November 2013) [121], National Center for Biotechnology Information non-redundant (NCBI-nr) (4 November 2013) [122], and M5 non-redundant (M5-nr) (27 January 2014) [123] databases for functional annotation using BLASTP (e-value cutoff 0.001) [38]. UniRef90 gene entries sharing the highest percent identity with the query and NCBI-nr and M5-nr entries with the highest bit score match to the query were retained for annotation. Genes predicted by two or more methods (redundant) were considered the same and collapsed into a single entry if they shared the same start and stop position, orientation, and similar functional annotations. Non-redundant entries (i.e., gene predictions unique to a given software) were also retained. Finally, the above predictions and annotations were reconciled with the genes predicted and annotated through the Integrated Microbial Genomes Expert Review (IMG-ER) pipeline [124]. Selected origins of replication were verified by Ori-Finder [125]. The genes in the genome was assigned taxa in the NCBI taxonomy based on the BLASTN [38] searches (-best_hit_overhang 0.25, -best_hit_score_edge 0.05, -evalue 0.0001) against the NCBI-nr database (8 July 2014) computed with MEGAN 5.4.3 (maximum number of matches per read 100; LCA parameters: minimal support 5, minimal score 35, top percent 10) [39]. Selected promoters were identified with BPROM [126]. Signal peptides and transmembrane domains were predicted using SignalP 3.0 Server and TMHMM, respectively [127]. The Genomic Utility for Automated Comparison (GUAC) Python script (Additional file 5) was developed to inform comparative analyses of gene content across multiple genomes, in particular genes involved in sulfur-oxidation (Figure 4) and extracellular transport (Table 2). The GUAC software first identified those target genes in the genomes of interest that were annotated with unambiguous gene symbols (e.g. *soxA*). Next, using amino acid sequences of these genes as queries, BLASTP searched for homologous sequences in the remaining target genomes (default cut-off values: bit score 50, identity 30%, alignment length over the source sequence 40%). These sequences were aligned using ClustalW [128]. The alignments were used to manually verify the results (e.g., based on known conserved domains, etc.). Mobile genetic elements were detected by type. Insertion sequences were found using OASIS [129]. Integrative conjugative elements and plasmid as well as phage sequences were identified by BLASTN [38] searches against the ICEberg [130] and ACLAME [131] databases, respectively (cut-off values: 250 nucleotides alignment length and 90% identity). To determine whether mobile genetic elements interrupted open reading frames, the nucleotide regions before and after each element were concatenated and aligned to the NCBI-nr sequences using BLASTN.

### Availability of supporting data

This genome project has been deposited at DDBJ/EMBL/GenBank under the accession JRAA00000000. The version described in this paper is version JRAA01000000.

## Electronic supplementary material

Additional file 1: Table S1: Length [bp], GC%, percentage of the total base pairs, and the number of genes in the scaffolds which constitute the genome of the *S. velum* symbiont. (XLSX 52 KB)

Additional file 2: Table S2: tRNA genes and the codon frequencies in the genome of the *S. velum* symbiont. (XLSX 44 KB)

Additional file 3: Table S3: Gene product names used in Figures 1 and 4, the corresponding NCBI protein ID reference numbers, and EC/TC numbers. (XLSX 76 KB)

Additional file 4: Table S4: Parameters of the gene prediction software. (XLSX 39 KB)

Additional file 5: **Genomic Utility for Automated Comparison (GUAC).** A Python script developed to inform comparative analyses of gene content across multiple genomes. (ZIP 5 KB)
